# Gene regulation in *Cryptosporidium*: New insights and unanswered questions

**DOI:** 10.1016/j.crpvbd.2025.100280

**Published:** 2025-06-17

**Authors:** Samantha Gunasekera, Jessica C. Kissinger

**Affiliations:** a Center for Tropical and Emerging Global Diseases, University of Georgia, Athens, GA, USA; b Department of Genetics, University of Georgia, Athens, GA, USA; c Institute of Bioinformatics, University of Georgia, Athens, GA, USA

**Keywords:** Polycistronic transcription, Epigenetics, Apicomplexa, Non-coding RNA, Transcription factor

## Abstract

Parasites of the genus *Cryptosporidium* have evolved to have a highly compact genome of ∼9.1 Mb. The mechanisms that regulate gene expression in *Cryptosporidium* spp. remain incompletely understood at all levels, including chromatin accessibility, transcription factor activation and repression and RNA processing. This review discusses possible mechanisms of gene regulation in *Cryptosporidium* spp., including histone modifications, *cis* regulatory elements, transcription factors and non-coding RNAs. *Cryptosporidium* spp. are among the most basal branching apicomplexans and existing evidence suggests that they diverge from other members of their phylum *via* retention of the E2F/DP1 transcription factor family, and the recent discovery that *C. parvum* produces polycistronic transcripts. Most of what we know about gene regulation in the genus *Cryptosporidium* is based on sequence conservation and homology with other members of the phylum Apicomplexa, and in some cases, more distant eukaryotes. Very few putative gene regulatory components identified in *Cryptosporidium* spp. are supported by experimental confirmation. This review summarizes what we know about gene regulation in *Cryptosporidium* spp. and identifies gaps in our current understanding.

## Highlights

• Most gene regulatory components in *Cryptosporidium* lack functional validation.
• Gene regulation in *Cryptosporidium* shares similarities with other apicomplexans.
• Two key differences specific to *Cryptosporidium* gene regulation are listed below:
• *Cryptosporidium* spp. are the only apicomplexans to have retained the E2F/DP1 transcription factor family.
• *C. parvum* is among the few eukaryotes to have evolved polycistronic transcription.

## Graphical abstract

**Figure:**
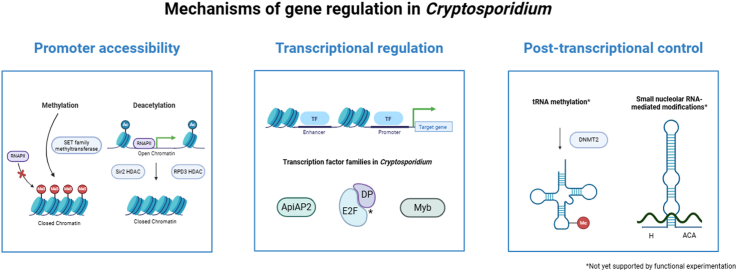
Created in BioRender. Gunasekera, S. (2025) https://BioRender.com/0p0fo7f.

## 1 Introduction

The transcriptional regulatory networks that control gene expression in *Cryptosporidium* spp. remain incompletely understood. Gene expression in *Cryptosporidium* spp. occurs in a tightly programmed cascade over the course of intracellular development, with distinct clusters of transcripts that rise and fall in abundance together as the parasite transitions through morphologically and functionally distinct life cycle stages (Mauzy et al., 2012; Oberstaller et al., 2013; Walzer et al., 2024). The prevailing dogma of apicomplexan gene regulation indicates that the production of transcripts occurs “just-in-time”, only if and when induction of the target gene is required (Bozdech et al., 2003). Synchronous gene expression patterns between co-regulated clusters of genes do not correlate with chromosomal location, with co-localized gene families often having vastly different expression profiles at different stages of the life cycle (Mauzy et al., 2012).

The genus *Cryptosporidium* is among the most basal-branching members of the phylum Apicomplexa and may provide insights into the ancestral mechanisms of gene regulation (Waller and Carruthers, 2024). Broadly, apicomplexans are well-documented to utilize an expansion of the apicomplexan AP2 (ApiAP2) family of transcription factors for gene regulation (Balaji et al., 2005), in the absence of many canonical eukaryotic transcription factors (Templeton et al., 2004). *Cryptosporidium* spp. are the only members of the phylum to have retained the E2F/DP1 transcription factor family, and is demonstrably less reliant on the ApiAP2 superfamily for gene regulation (Oberstaller et al., 2013).

In eukaryotes, the accessibility of *cis* regulatory elements by DNA binding proteins is controlled by chromatin structure, which is highly dynamic and regulated by a suite of histone-modifying enzymes and ncRNAs. Additionally, while most eukaryotes encode just one gene per mRNA molecule, polycistronic transcripts have been recently discovered in *Cryptosporidium parvum* sporozoites and merozoites (Xiao et al., 2025); a first for the Apicomplexa.

In this review, we will examine existing lines of scientific inquiry suggesting that *Cryptosporidium parvum* utilizes transcriptional regulatory systems that diverge somewhat from the model of gene regulation in other apicomplexans. We aim to provide an overview of what is already known about gene regulation in *Cryptosporidium* spp., and what remains to be elucidated.

## 2 Epigenetic regulation of gene expression

### 2.1 Histone tail post-translational modifications

**Fig. 1:**
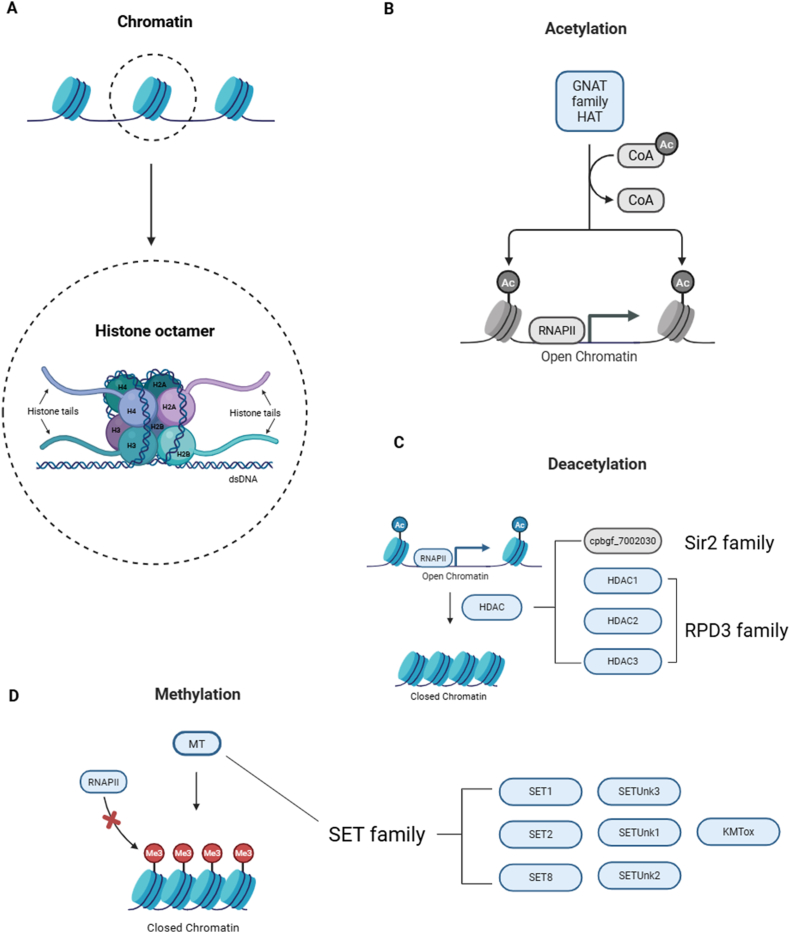
Enzymes in *C. parvum* with possible roles in post-translational modifications of histone tails. **A** Schematic diagram of chromatin and histone octamer structure. **B***Cryptosporidium parvum* does possess GNAT family HAT orthologs but the HAT pathway has been shaded grey since none have been functionally confirmed. **C***Cryptosporidium parvum* encodes three RPD3 family HDACs which have been demonstrated to bind histone tails, and one Sir2 family HDAC that has not been experimentally confirmed (shown in grey). **D** Seven out of eight SET family proteins in *C. parvum* were identified as putative histone methyltransferases (Sawant et al., 2022). Created in Biorender. Gunasekera, S. (2025) https://BioRender.com/wp3u43V.

In eukaryotes, nucleosomes are the fundamental organizational unit of chromatin. Each nucleosome is comprised of a histone octamer containing two copies each of the H2A, H2B, H3, and H4 histone core proteins wrapped with ∼147 bp of DNA (Fig. 1A) and is separated by 10–90 bp of linker DNA often bound by linker histones (Richmond and Davey, 2003). Nucleosomes are intrinsically dynamic, and function to regulate access to DNA by other nuclear factors. The presence of nucleosomes predates the divergence of eukaryotes and archaea in evolution, indicating that the role of chromatin architecture in gene regulation is ancient (Ammar et al., 2012). Linker histones are non-essential in lower organisms, and the *C. parvum* reference genome assembly has no annotated linker histone protein encoding genes (Abrahamsen et al., 2004).

Histone core proteins each have an N-terminal histone tail domain and a C-terminal histone fold domain, though H2A monomers each possess an additional C-terminal histone tail. The histone tails are positively charged, facilitating the association with negatively charged DNA (Sterner and Berger, 2000). Histone tails are rich in lysine and arginine residues that are often the site of post-translational modifications, which can include acetylation, phosphorylation, methylation, ubiquitination, ADP-ribosylation, biotinylation, and lactylation, though this list is not exhaustive (Hansen, 2002). Different combinations of post-translational modifications mark functional units of chromatin that recruit proteins that can activate or repress gene expression, regulate DNA replication and chromatin remodelling. These post-translational modifications are collectively referred to as the histone code (Strahl and Allis, 2000). Epigenetic regulation of gene expression in *Cryptosporidium* spp. is under-studied, and only a subset of post-translational modifications of histone tails have been investigated.

#### 2.1.1 Acetylation and deacetylation

Histone acetyltransferases (HATs) and deacetylases (HDACs) play key roles in transcriptional regulation (Strahl and Allis, 2000). HATs transfer an acetyl group from acetyl-CoA to a lysine residue in a histone tail, partially neutralizing its positive charge and weakening the histone-DNA association. The outcome is increased accessibility of a locus to transcriptional activation machinery. The removal of the acetyl group catalyzed by HDACs is associated with transcription repression. HATs and HDACs work synergistically to regulate transcription (Sterner and Berger, 2000).

Most HATs belong to the ancient GNAT superfamily and the MYST family (Iyer et al., 2008). The *C. parvum* reference genome has several annotated GNAT family acetyltransferases (Abrahamsen et al., 2004), though none have been the subject of functional investigation (Fig. 1A). No MYST family HATs are annotated in the *C. parvum* reference genome (Abrahamsen et al., 2004), though MYST protein domains are present in more recent *Cryptosporidium* spp. genome assemblies (Baptista et al., 2022). The HDACs are comparatively better studied in *Cryptosporidium* spp. (Fig. 1B) and can be divided into three structurally unrelated groups, the HD2 family, Sir2 family, and the RPD3 family. The HD2 family is unique to plants, while the Sir2 and RPD3 are superfamilies present across eukaryotes, prokaryotes and archaea (Iyer et al., 2008; Rider and Zhu, 2009). The RPD3 superfamily uses metal-dependent catalysis, and the Sir2 superfamily uses a NAD cofactor (Iyer et al., 2008). *Cryptosporidium parvum* possesses three RPD3 family HDAC proteins (Table 1) that are postulated to play a role in regulation of DNA replication (Rider and Zhu, 2009), and one putative Sir2 family protein (cpbgf_7002030/cgd7_2030) that has limited *C. parvum*-specific functional information available (Yasukawa and Yagita, 2010).

**Table 1:** RPD3 family HDACs in *Cryptosporidium parvum*.

Gene ID	Protein name^a^	Predicted target^a^	Life cycle stage^b^	Single-cell atlas designation^b^
cpbgf_60080/cgd6_80	HDAC1	–	Asexual	Cluster 4
cpbgf_6001380/cgd6_1380	HDAC2	–	Not stage-specific	Clusters 3–5, 14, 15
cpbgf_800480/cgd8_480	HDAC3	H4K8, H4K12	Not stage-specific	Clusters 2, 6, 7, 10, 11, 14–17

a The RP3 family HDACs in *C. parvum* were originally characterized by Rider and Zhu (2009).

b Single-cell transcriptome atlas designation and life cycle stage(s) of expression from Walzer et al. (2024). Clusters 1–9 correspond to asexual development, 10–12 correspond to male sexual development, and 13–18 correspond to female sexual development.

#### 2.1.2 Methylation and demethylation

Histone methyltransferases add methyl groups to lysine residues in histone tail domains, increasing the basicity of the lysine. Methylation of histone lysine residues in eukaryotes is most commonly catalyzed by methyltransferases in the SET domain superfamily (Fig. 1C), though it can also be catalyzed by the DOT1 family which lack SET domains (Khorasanizadeh, 2004). Lysine methylation predominantly occurs on histone H3 tails, and methylation of H3K4, H3K79, and H3K36 is associated with gene activation, while methylation of H3K9, H3K27, and H4K20 is associated with repressed gene expression (Sautel et al., 2007). Demethylation is carried out using Jumonji-related domain containing proteins (JmjC) (Iyer et al., 2008). *Cryptosporidium parvum* encodes several SET domain-containing proteins (Table 2) but does not encode any JmjC domain containing proteins (Sawant et al., 2022). Gastric species of *Cryptosporidium* including *C. andersoni* and *C. muris* encode a DOT1 family methyltransferase that is absent in *C. parvum* (Sawant et al., 2022).

**Table 2:** SET domain-containing proteins in *Cryptosporidium parvum*.

Gene ID	Protein name^a^	Predicted target^a^	Life cycle stage^b^	Single cell atlas designation^b^
cpbgf_8002730/cgd8_2730	SET1	H3K4	Asexual	Clusters 3, 4, 10, 13
cpbgf_500400/cgd5_400	SET2	H3K36	Not stage-specific	Clusters 2, 3, 10, 13
cpbgf_400370/cgd4_370	SET8	H4K20	Not stage-specific	–
cpbgf_4002090/cgd4_2090	AKMT	Possible non-histone methyltransferase	Sexual	Clusters 7, 8
cpbgf_5002340/cgd5_2340	KMTox	H4/H2A	Not stage-specific	–
cpbgf_7005090/cgd7_5090	SET Unk1	H3K4	Asexual	–
cpbgf_1002170/cgd1_2170	SET Unk2	H3K4	Asexual	–
cpbgf_6001470/cgd6_1470	SET Unk3	H3K27	Asexual	Cluster 2

a The SET domain family methyltransferases in *C. parvum* were originally characterized by Sawant et al. (2022).

b Single-cell transcriptome atlas designation and life cycle stage where each protein was highly expressed was originally published by Walzer et al. (2024). Clusters 1–9 correspond to asexual development, 10–12 correspond to male sexual development, and 13–18 correspond to female sexual development.

#### 2.1.3 Lactylation

Lactylation of histones was recently detected in *Plasmodium falciparum* as an additional mechanism of post-translational histone modification potentially involved in epigenetic regulation (Merrick, 2023). In lactylation, a lactyl group is added to lysine residues in histones. Lactylation of histones is generally considered to be a mark of activation and was only recently discovered in humans (Zhang et al., 2019). In humans, and in *P. falciparum*, lactylation levels fluctuate with lactate levels, providing potential environmental cues. In *P. falciparum*, hyperlactatemia is a strong predictor of severe malarial disease (Possemiers et al., 2021). *Cryptosporidium parvum* does contain a bacterial-type cytosolic and parasitophorous vacuole membrane associated L-lactate dehydrogenase (LDH) that can produce lactate (Zhang et al., 2015). Currently, it is unknown if lactylation of histones exists in *C. parvum* and thus may play a role in epigenetic regulation.

### 2.2 Chromatin remodelling

Enzymes involved in chromatin remodelling typically utilize NTP hydrolysis and usually contain P-loop NTPase folds (Iyer et al., 2008). There are two main classes of these enzymes: SWI2/SNF2 ATPases which perform local remodelling by altering nucleosome positioning, and the SMC ATPases which belong to the ABC superfamily (Iyer et al., 2008). The *C. parvum* reference genome has several annotated SWI2/SNF2 and SMC ATPases that possibly have roles in chromatin remodelling but have not been the subject of functional investigation. Some Myb domain containing proteins may have roles in chromatin remodelling (see *Section* 3.3); however, these predictions are based on homology to proteins in other organisms and lack functional confirmation in *Cryptosporidium* spp.

### 2.3 Cytosine methylation

In higher eukaryotes, CpG dinucleotides are often a target for covalent attachment of methyl groups, where the methyl group protrudes from the cytosine nucleotide into the major groove. The effect is two-pronged; transcription factors are displaced, and the attraction of methyl-binding proteins is associated with gene silencing and chromatin compaction (Fazzari and Greally, 2004). Cytosine-5 DNA methyltransferases catalyze the attachment of methyl groups to cytosine, and this family of enzymes is conserved in most eukaryotes. *Cryptosporidium parvum* encodes one annotated cytosine-5 DNA methyltransferase, DNMT2 (cpbgf_5002100/cgd5_2100), which is most likely involved in RNA modifications (see *Section* 4.2). In some eukaryotes, cytosine methylation is not always localized to CpG dinucleotides (Fisher et al., 2004), though genome-wide assessments using several detection methods have demonstrated that *C. parvum* has no detectable cytosine methylation (Gissot et al., 2008). While this trait was initially thought to be shared with *Toxoplasma gondii* and *P. falciparum* (Choi et al., 2006; Gissot et al., 2008), more recent investigations demonstrated low levels of cytosine methylation in *P. falciparum* (Gissot et al., 2008; Lucky et al., 2023) and *T. gondii* (Wei et al., 2017). Cytosine methylation in DNA is not considered a major contributor to epigenetic processes in *Cryptosporidium* spp.

## 3 Transcription factors

### 3.1 ApiAP2 family

**Fig. 2:**
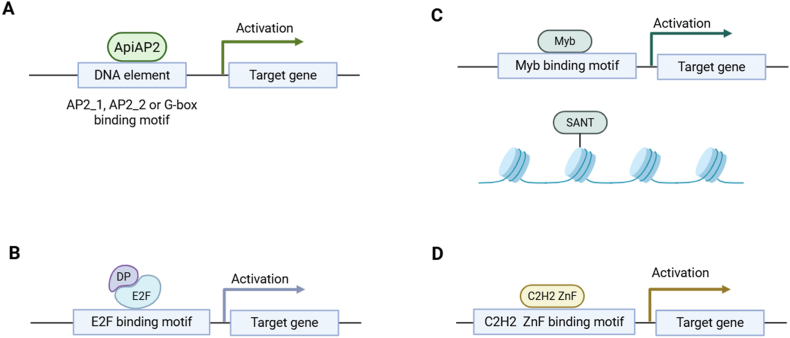
Possible transcription factors in *C. parvum* that may have a role in gene regulation. Little is known about the entire transcription factor complexes that regulate gene expression in *Cryptosporidium* spp. Schematic diagrams representing our understanding of ApiAP2 transcription factor activation of gene expression in *Cryptosporidium* spp. (**A**), E2F/DP transcription factor activity in eukaryotes (**B**), possible Myb/SANT gene regulation in *Cryptosporidium* spp. (**C**), and possible role of C2H2 ZnF in *Cryptosporidium* spp. gene regulation (**D**). Only one transcription factor protein for each scenario is shown but several may be present. Created in BioRender. Gunasekera, S. (2025) https://BioRender.com/odz641n.

The apicomplexan AP2 (ApiAP2) transcription factor family members are the most extensively studied gene regulatory components across the Apicomplexa (Fig. 2A). Structurally, the AP2 protein domains that define the ApiAP2 transcription factors are ∼60 amino acids in length, with three highly conserved β-strands followed by a less strongly conserved α-helix (Balaji et al., 2005; De Silva et al., 2008). There are 18 proteins encoded by the *C*. *parvum* genome that are predicted to contain at least one AP2 domain (Oberstaller et al., 2014). ApiAP2 protein domains in *C. parvum* have been experimentally confirmed to bind a range of DNA motifs summarized in Table 3. While the ApiAP2 family of transcription factors resemble the Apetala 2/Ethylene Responsive Factor (AP2/ERF) group of transcription factors found in plants, there are many additional family members in apicomplexans. There is also greater sequence diversity in the ApiAP2 transcription factor family, and consequently a wider repertoire of DNA motifs that they will bind (Balaji et al., 2005). Phylogenetic analyses of AP2 domain sequences across the SAR clade (Burki et al., 2020), encompassing the Stramenopila, Alveolata and Rhizaria lineages (Grattepanche et al., 2018), have indicated that the AP2 domains in apicomplexans are more closely related to the AP2 domains in Perkinsozoa than the Dinoflagellata (Oberstaller et al., 2014). There are four AP2 domains across three *C. parvum* proteins that likely existed in the myzozoan common ancestor, though lost in the dinoflagellates (Oberstaller et al., 2014). Ten AP2 domains across nine *C. parvum* proteins have homologs in most apicomplexans but are absent outside of the phylum, and ten AP2 domains across eight *C. parvum* proteins are specific to *C. parvum* (Oberstaller et al., 2014), indicating that most of their ApiAP2 transcription factors are the result of lineage-specific expansions, which is also the case for the genera *Plasmodium* and *Toxoplasma* (Balaji et al., 2005). Within the genus *Cryptosporidium*, most AP2 domains are conserved, but there is variation between species. For example, *C. muris* and *C. andersoni* are each missing five domains and additionally have two differences between them. Domain confirmation in all species will require gapless genome assemblies.

**Table 3:** List of overrepresented putative DNA-binding motifs in *Cryptosporidium parvum* upstream promoter regions.

Motif family	Motif sequence	Reference	*Trans* factor
AP2_1	5′-TGCATGCA-3′	Bankier et al. (2003); Oberstaller et al. (2013)	ApiAP2 (Table 4)
AP2_2	5′-GCACAC-3′	Oberstaller et al. (2013)	ApiAP2 (Table 4)
G-box	5′-G.GGGG-3′	Mullapudi et al. (2007); Cohn et al. (2010); Oberstaller et al. (2013)	ApiAP2 (Table 4)
E2F	5′-[A/T][C/G]GCGC[G/C][A/T]-3′	Bankier et al. (2003); Oberstaller et al. (2013)	E2F/DP (Table 5)
GAGA	5′-GAGAGAGA-3′	Oberstaller et al. (2013)	Unknown
CAAT-box	5′-GGCCAATCT-3′	Oberstaller et al. (2013)	bZIP (not reviewed here)

Expression of many members of the ApiAP2 transcription factor family in *C. parvum* appears to be stage-specific (Table 4), and there is considerable evidence indicating that they may play a large role in male and female fate determination (Tandel et al., 2019, 2023; Hasan et al., 2024; Walzer et al., 2024). The regulatory targets of most of the ApiAP2 transcription factors remain unknown, except for AP2-F, which has been demonstrated to regulate the expression of six proteins of the crystalloid body (cgd8_4290, cgd7_5140, cgd7_300, cgd7_1730, cgd2_790, cgd2_2110) and cgd7_5050 (NIMA kinase) (Tandel et al., 2023).

**Table 4:** List of ApiAP2 domain-containing proteins in *Cryptosporidium parvum.*

Gene ID^a^	Life cycle stage	Single-cell atlas designation^b^	Primary binding motif	Evolutionary clade^a^
cpbgf_8003230/cgd8_3230	Asexual (Tandel et al., 2019; Walzer et al., 2024)	Cluster 1	AP2_1-like (Oberstaller et al., 2013, 2014)	Ancestral
cpbgf_400600/cgd4_600	Not stage-specific (Tandel et al., 2019; Walzer et al., 2024)	Cluster 6	AP2_2-like (Oberstaller et al., 2013, 2014)	Pan-apicomplexan
cpbgf_5002570/cgd5_2570	Asexual (Walzer et al., 2024), *in vivo* female (Tandel et al., 2019)	Clusters 6, 7	5′-GTGTGT-3′ (Oberstaller et al., 2014)	Pan-apicomplexan
cpbgf_8003130/cgd8_3130	Asexual (Mauzy et al., 2012; Walzer et al., 2024), *in vivo* female (Tandel et al., 2019)	Cluster 7	AP2_2-like (Oberstaller et al., 2013, 2014)	Ancestral
cpbgf_1003520/cgd1_3520	Not stage-specific (Walzer et al., 2024)	Cluster 7	AP2_1-like (Oberstaller et al., 2013, 2014)	Pan-apicomplexan
cpbgf_4002950/cgd4_2950	Asexual (Mauzy et al., 2012; Walzer et al., 2024), *in vivo* female (Tandel et al., 2019)	Cluster 8	5′-GCGTGCA-3′ (Oberstaller et al., 2014)	*Cryptosporidium*-specific
cpbgf_3002970/cgd3_2970	Male-specific (Walzer et al., 2024)	Clusters 10, 11	Does not bind DNA (Oberstaller et al., 2014)	*Cryptosporidium*-specific
cpbgf_6002670/cgd6_2670 (AP2-M)	Male-specific (Tandel et al., 2023; Walzer et al., 2024)	Cluster 11	5′-AAAA-3′ (Oberstaller et al., 2014)	*Cryptosporidium*-specific
cpbgf_2003490/cgd2_3490	Female-specific (Mauzy et al., 2012; Tandel et al., 2019; Hasan et al., 2024; Walzer et al., 2024)	Clusters 15-18	AP2_1-like (Oberstaller et al., 2013, 2014)	Pan-apicomplexan
cpbgf_4001110/cgd4_1110 (AP2-F)	Female-specific (Mauzy et al., 2012; Tandel et al., 2019, 2023; Walzer et al., 2024)	Clusters 16, 17	AP2_1-like (Oberstaller et al., 2013, 2014)	Ancestral
cpbgf_800810/cgd8_810	Female-specific (Tandel et al., 2019; Walzer et al., 2024)	Clusters 16-18	G-box (Oberstaller et al., 2013, 2014)	Pan-apicomplexan
cpbgf_6005320/cgd6_5320	Not stage-specific (Tandel et al., 2019; Walzer et al., 2024)	–	Does not bind DNA (Oberstaller et al., 2014)	Pan-apicomplexan
cpbgf_3001980/cgd3_1980	Not stage-specific (Tandel et al., 2019; Walzer et al., 2024)	–	Does not bind DNA (Oberstaller et al., 2014)	Pan-apicomplexan
cpbgf_5004250/cgd5_4250	Not stage-specific (Tandel et al., 2019; Walzer et al., 2024)	–	AP2_1-like (Oberstaller et al., 2013, 2014)	Pan-apicomplexan
cpbgf_6001140/cgd6_1140	Not stage-specific (Walzer et al., 2024)	–	Does not bind DNA (Oberstaller et al., 2014)	*Cryptosporidium*-specific
cpbgf_4003820/cgd4_3820	Not stage-specific (Walzer et al., 2024)	–	AP2_1-like (Oberstaller et al., 2013, 2014)	Pan-apicomplexan
cpbgf_6002600/cgd6_2600	Not stage-specific Tandel et al. (2019)	–	5′-GTGTGT-3′ (Oberstaller et al., 2014)	*Cryptosporidium*-specific
cpbgf_2002990/cgd2_2990	–	–	G-box (Oberstaller et al., 2014)	*Cryptosporidium*-specific

a Identification of ApiAP2 domain-containing proteins and kingdom-wide evolutionary analysis of AP2 domains was originally published by Oberstaller et al. (2014).

b Single-cell transcriptome atlas designation was originally published by Walzer et al. (2024). Clusters 1–9 correspond to asexual development, 10–12 correspond to male sexual development, and 13–18 correspond to female sexual development.

### 3.2 E2F family

*Cryptosporidium* thus far, appears to be the only genus in the Apicomplexa to have retained the E2F/DP transcription factor family (Templeton et al., 2004; Oberstaller et al., 2013; Baptista et al., 2025). The E2F transcription factor family comprises two subfamilies: E2F and DP. In higher eukaryotes, one member of each of these subfamilies come together to form an active heterodimer that binds promoters to regulate the expression of many target genes, where they can act as either transcription activators or repressors (Fig. 2B). E2F transcription activity in higher eukaryotes can be modulated through complex formation with other regulatory proteins (Attwooll et al., 2004). The *C. parvum* genome encodes two E2F transcription factors and two DP binding partners (Table 5), which peak in expression at 2 h and 12 h post-infection (Mauzy et al., 2012). It remains unknown whether *C. parvum* encodes any other proteins that interact with the E2F transcription factors, or how they may utilize E2F transcription factors for gene regulation. Of interest, E2F motifs are the most abundant transcription factor binding site in *C. parvum* (Oberstaller et al., 2013) and have been recently suggested to be an important regulator of early asexual development and polycistronic transcripts (Xiao et al., 2025). E2F motifs are overrepresented upstream of genes associated with DNA replication and glycolysis (Oberstaller et al., 2013), and in the internal transcribed spacer of genes present in polycistronic transcripts (Xiao et al., 2025). E2F motifs are also overrepresented upstream of co-regulated ribosomal protein encoding genes in *C. parvum*, starkly differing from the G-box motif found in other Apicomplexa (Oberstaller et al., 2013), lending further support to extensive differences in gene regulation mechanisms in the genus *Cryptosporidium*.

**Table 5:** List of E2F/DP transcription factors in *Cryptosporidium parvum.*

Gene ID	Protein subfamily	Life cycle stage	Single-cell atlas designation^a^	Binding motif^b^
cpbgf_1001560/cgd1_1560	E2F	Asexual	Clusters 1, 2	5′-[A/T][C/G]GCGC[G/C][A/T]-3′
cpbgf_6001430/cgd6_1430	E2F	Asexual	Clusters 1, 2	5′-[A/T][C/G]GCGC[G/C][A/T]-3′
cpbgf_7003650/cgd7_3650	DP	Asexual	Clusters 1, 2	5′-[A/T][C/G]GCGC[G/C][A/T]-3′
cpbgf_8001850/cgd8_1850	DP	Asexual	Clusters 1, 2	5′-[A/T][C/G]GCGC[G/C][A/T]-3′

a Single-cell transcriptome atlas designation was originally published by Walzer et al. (2024), and mined for E2F/DP expression by Xiao et al. (2025).

b The E2F binding motif in *C. parvum* was originally reported by Bankier et al. (2003) and investigated further by Oberstaller et al. (2013).

### 3.3 Myb transcription factors

The Myb transcription factor superfamily is defined by the Myb DNA binding domain that contains between one to four Myb repeats stretching 50–53 amino acids in length each, termed R1, R2, R3, and R4. Each Myb repeat contains three alpha helices, the second and third of which confers the helix-turn-helix secondary structure that enables DNA binding. The Myb superfamily is subdivided into four groups (1R-Myb, 2R-Myb, 3R-Myb, and 4R-Myb) based on the number of Myb repeats it possesses and their position within the Myb domain. Importantly, a subgroup of 1R- and 2R Mybs termed the SANT domain proteins cannot bind DNA and instead interact with histone tails (Fig. 2C), often as part of multimeric protein complexes (Prouse and Campbell, 2012). The *C. parvum* genome has 16 annotated Myb domain-containing proteins (Table 6). The diverse roles of Mybs in apicomplexans have been recently reviewed comprehensively (Schwarz and Lourido, 2023). The only Myb in *C. parvum* that has been functionally validated is Myb-M (cpbgf_6002250/cgd6_2250), which is the earliest transcription factor that controls male sexual fate determination in *C. parvum* (Walzer et al., 2024).

**Table 6:** Myb domain-containing proteins in *Cryptosporidium parvum*.

Gene ID^a^	Suggested role in gene regulation^a^	Myb subfamily^a^	Life cycle stage^b^	Single cell atlas designation^b^
cpbgf_6004510/cgd6_4510	DNA binding	1R-Myb	Not stage-specific	Clusters 3, 4, 10
cpbgf_3002510/cgd3_2510	DNA binding	1R-Myb	Not stage-specific	–
cpbgf_5001120/cgd5_1120	Histone modification	1R-SANT	Not stage-specific	–
cpbgf_4001270/cgd4_1270	Histone modification	1R-SANT	Not stage-specific	–
cpbgf_8004840/cgd8_4840	–	–	Asexual	Cluster 1
cpbgf_1002330/cgd1_2330	Histone modification	1R-SANT	Not stage-specific	Clusters 4–6, 10–12
cpbgf_8002770/cgd8_2770	Chromatin remodelling	ISW1	Female-specific	Cluster 18
cpbgf_6003860/cgd6_3860	Chromatin remodelling	ISW1	Male-specific	Cluster 10
cpbgf_500110/cgd5_110	DNA binding	CDC5L	Not stage-specific	Clusters 3, 7, 11, 13
cpbgf_6002250/cgd6_2250 (Myb-M)	Male sexual fate determination	1-3R-Myb	Male-specific	Clusters 10, 11
cpbgf_2003980/cgd2_3980	Histone modification	1R-SANT	Not stage-specific	Clusters 8, 14
cpbgf_2002260/cgd2_2260	Displacement of polycomb-repressive complex	DNAJC	Asexual	Clusters 1, 9
cpbgf_2003460/cgd2_3460	–	CHY	Not stage-specific	–
cpbgf_2001740/cgd2_1740	Chromatin remodelling	SWI3	Not stage-specific	Clusters 3, 4, 10, 13, 14
cpbgf_400880/cgd4_880	Histone acetyltransferase	Ada2	Not stage-specific	Clusters 9, 12
cpbgf_3001120/cgd3_1120	DNA binding	4R-Myb	Not stage-specific	Clusters 9, 11

a Myb domain-containing proteins in the Apicomplexa have been reviewed in detail by Schwarz and Lourido (2023). Note that suggested roles in gene regulation are based on sequence homology only and have not been functionally investigated.

b Single-cell transcriptome atlas designation and the highest expressed life cycle stage was originally published by Walzer et al. (2024). Clusters 1–9 correspond to asexual development, 10–12 correspond to male sexual development, and 13–18 correspond to female sexual development.

### 3.4 C2H2 zinc finger proteins

Zinc finger domains (ZnF) are among the most widespread DNA binding domains in eukaryotes. They bind zinc ions most commonly *via* pairs of cysteine and histidine residues (C2H2 ZnF). C2H2 ZnF have a beta-beta-alpha secondary structure, and the basic and hydrophobic residues in the alpha helix confer DNA binding capability. A single C2H2 ZnF cannot typically function alone to regulate transcription, and typically a series of three or more C2H2 ZnF will bind *cis* regulatory elements (Matthews and Sunde, 2002). While *C. parvum* encodes several putative C2H2 ZnFs (Table 7), their DNA binding sites and their role in gene regulation in *Cryptosporidium* spp. remains uncharacterized (Fig. 2D).

**Table 7:** Annotated C2H2 ZnF domain-containing proteins in *Cryptosporidium parvum*.

Gene ID^a^	Life cycle stage^b^	Single cell atlas designation^b^
cpbgf_2001150/cgd2_1150	Not stage-specific	Clusters 2, 3, 10, 13
cpbgf_3001060/cgd3_1060	Not stage-specific	–
cpbgf_3001440/cgd3_1440	Not stage-specific	–
cpbgf_5001110/cgd5_1110	Not stage-specific	–
cpbgf_7001170/cgd7_1170	Not stage-specific	–
cpbgf_7004300/cgd7_4300	Asexual	Clusters 8, 9
cpbgf_7005380/cgd7_5380	Not stage-specific	Clusters 3–5, 10, 13–15
cpbgf_8001550/cgd8_1550	Asexual	Clusters 1, 6

a C2H2 ZnF domain-containing proteins were mined from the updated *C. parvum* genome annotation in Baptista et al. (2025).

b Single-cell transcriptome atlas designation and the highest expressed life cycle stage was originally published by Walzer et al. (2024). Clusters 1–9 correspond to asexual development, 10–12 correspond to male sexual development, and 13–18 correspond to female sexual development.

## 4 Non-coding RNAs

There are several types of non-coding RNAs (ncRNAs) and many play roles in gene expression at the level of epigenetics, transcription, transcript processing and translation. The role of ncRNAs in apicomplexans has been reviewed in detail elsewhere (Li et al., 2020; Mitesser et al., 2024). In *C. parvum*, ncRNAs are abundant (Li et al., 2021, 2022), and approximately 10% of the genes in *C. parvum* have an associated antisense transcript of unknown function (Li et al., 2021; Baptista et al., 2025).

### 4.1 Long non-coding RNAs

Long non-coding RNAs (lncRNAs) are defined as transcripts longer than 200 nucleotides with open reading frames shorter than 30 amino acids that do not encode a known protein product. Several ncRNAs, especially lncRNAs, have been well characterized in the apicomplexan parasite genera *Plasmodium* and *Toxoplasma*, where they have been implicated in epigenetic regulation of numerous genes and processes, including *var* gene expression (Jing et al., 2018), telomere-associated repetitive elements (TARE) (Broadbent et al., 2011), and translational blocking (Eksi et al., 2012). In *C. parvum*, numerous lncRNAs have been annotated and studied for patterns of developmentally regulated gene expression (Li et al., 2021; Baptista et al., 2025). In the *C. parvum* IOWA II BGF Telomere-to-Telomere genome assembly (Baptista et al., 2025), 766 lncRNAs have been annotated based on gene expression data. Natural antisense transcripts are a subtype of lncRNA that either partially or entirely, overlap a corresponding sense transcript. They have been reported extensively in *P. falciparum* and *T. gondii* (Radke et al., 2005; Siegel et al., 2014), though their function is not well-understood. Existing evidence indicates that most annotated lncRNAs in *C. parvum* fall under the definition of a natural antisense transcript (Li et al., 2021). Given that *C. parvum* lacks genes that encode Dicer and Argonaute (Abrahamsen et al., 2004), natural antisense transcripts likely exert their influence on gene regulation in ways other than triggering dsRNA degradation (Abrahamsen et al., 2004). The only functionally characterized lncRNAs in *C. parvum* are those that are exported and influence host gene expression (Wang et al., 2016, 2017; Ming et al., 2017).

### 4.2 Small ncRNAs and epitranscriptomics

**Fig. 3:**
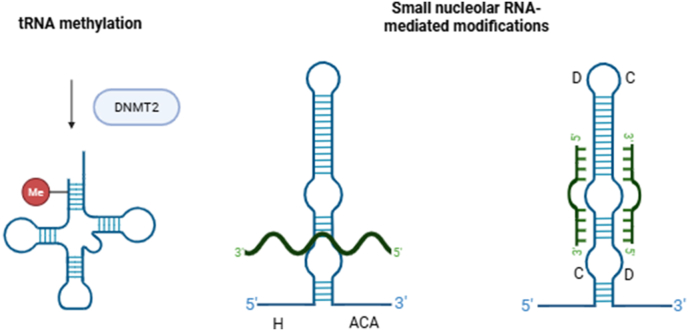
Possible epitranscriptomic modifications that *Cryptosporidium* spp. could utilize for gene regulation. Small RNAs are shown in blue, methyl groups are shown in red, modified regions of target RNAs are shown in green. Created in BioRender. Gunasekera, S. (2025) https://BioRender.com/9bqfw87.

Very little is known about RNA modifications in the genus *Cryptosporidium*, especially those that may affect gene expression (Fig. 3). We have non-validated evidence of RNA modifications in *C. parvum* based on Oxford Nanopore Direct RNAseq data, but the exact chemical modifications are computational predictions at this time (unpublished data). One gene encoding an ortholog of the methyltransferase DNMT2 protein (cpbgf_5002100/cdg5_2100), is annotated in the *C. parvum* reference genome (Abrahamsen et al., 2004; Baptista et al., 2025). DNMT2 is involved in tRNA methylation and possibly other RNA modifications (Lucky et al., 2023) and has been studied in other apicomplexans but not in *Cryptosporidium* spp.

Pseudouridinylation is a common post-transcriptional modification of RNA molecules in eukaryotes that results in the isomerization of uridine to pseudouridine. Pseudouridinylation can be catalyzed by ribonucleoproteins (RNPs) containing small nucleolar RNAs (snoRNAs) with H/ACA boxes that direct the position of pseudouridinylation in the RNA molecule (Charpentier et al., 2005). Pseudouridinylation in *T. gondii* has been functionally investigated and found to be developmentally regulated and to have a small but statistically significant effect on mRNA stability (Nakamoto et al., 2017). *Cryptosporidium parvum* contains orthologs of many genes encoding proteins in the H/ACA RNP complex as well as four H/ACA box snoRNAs (Table 8). The 2′-O-methylation of rRNA molecules can also be catalyzed by RNPs containing snoRNAs, with the exception that the snoRNAs contain C/D boxes (SNORDs) that direct the position of the post-transcriptional modification (Charpentier et al., 2005). *Cryptosporidium parvum* contains 5 identified C/D box snoRNAs (Table 8). The biological significance and modification targets in *Cryptosporidium* spp. are not experimentally confirmed for either class of modifications.

**Table 8:** List of H/ACA RBPs and SNORDs in *Cryptosporidium parvum*.

Gene ID	Modification/function
cpbgf_100530/cgd1_530	H/ACA ribonucleoprotein complex subunit Nop10
cpbgf_5001760/cgd5_1760	H/ACA ribonucleoprotein complex subunit
cpbgf_8001060/cgd8_1060	H/ACA ribonucleoprotein complex subunit Gar1/Naf1
cpbgf_5001585/cgd5_1585	H/ACA snoRNA
cpbgf_7002033/cgd7_2033	H/ACA snoRNA
cpbgf_7003425/cgd7_3425	H/ACA snoRNA
cpbgf_7005104/cgd7_5104	H/ACA snoRNA
cpbgf_2003583/cgd2_3583	C/D snoRNA
cpbgf_5003935/cgd5_3935	C/D SNORD96
cpbgf_6003219/cgd6_3219	C/D snoRNA
cpbgf_7001545/cgd7_1545	C/D snoRNA
cpbgf_7005107/cgd7_5107	C/D SNORD36

## 5 Upstream open reading frames

**Fig. 4:**
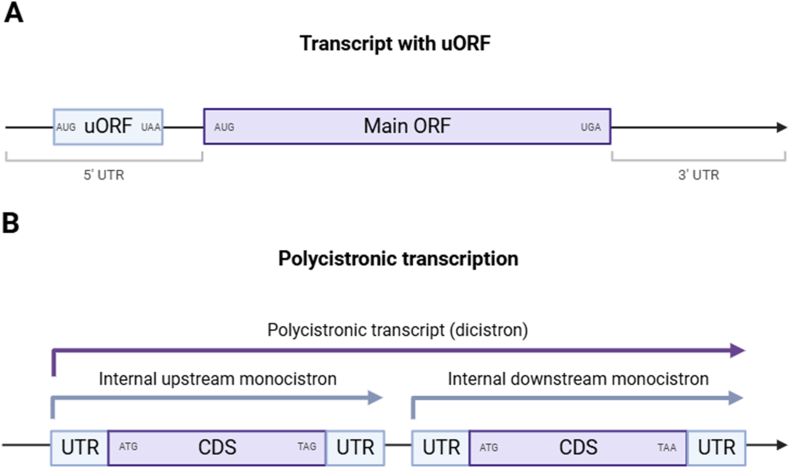
The structure of uORFs and polycistronic transcripts. Schematic diagram of a transcript with an uORF (not yet reported in *Cryptosporidium* spp.) (**A**), and a polycistronic transcript, recently reported in *C. parvum* (Xiao et al., 2025) (**B**). Created in BioRender. Gunasekera, S. (2025) https://BioRender.com/Uizemxv.

Translation in eukaryotes is initiated by the translation pre-initiation complex, which is recruited to the 5′ cap of the mRNA molecule before scanning downstream for an AUG start codon. Recognition of the start codon is heavily influenced by the surrounding Kozak sequence. In some organisms, the translation pre-initiation complex can also be recruited independently of the 5′ cap at internal ribosomal entry sites (IRES) (Karginov et al., 2017). Furthermore, translation initiation can be affected by the presence of an upstream open reading frame (uORF) where the 5′ untranslated region of an mRNA molecule also contains a start codon, with an in-frame stop codon that can be either upstream or within the main CDS (Fig. 4A). The presence of an uORF usually represses translation of the main CDS by either reducing efficiency of translation initiation at the main AUG or by triggering mRNA decay. Leaky ribosomal scanning can also occur, where the first translation initiation site may have a weaker Kozak sequence, resulting in the translation pre-initiation complex bypassing the first AUG (Dueñas et al., 2025). The presence of uORFs has been detected at an unusually high frequency in the genera *Toxoplasma* and *Plasmodium*, where almost all *Toxoplasma* and *Plasmodium* transcripts with an annotated 5′ UTR contain a uORF, reviewed in detail elsewhere (Kaur and Patankar, 2021). The presence of uORFs has not been described in *Cryptosporidium* spp., though future investigations are aided by the recent *C. parvum* IOWA-BGF Telomere-to-Telomere assembly with updated UTR annotations (Baptista et al., 2025).

The recent discovery of polycistronic transcripts in *C. parvum* sporozoites may represent a potentially related gene regulatory phenomenon where, instead of a short uORF being present in the 5′ UTR, an entire protein-encoding gene of unrelated function is transcribed upstream of a second ORF (Fig. 4B). The polycistronic transcripts most commonly consist of two genes and are termed dicistrons, but tri- and quad-cistronic genes are detected and were confirmed with RT-PCR. To date, 201 polycistronic transcripts have been detected in two different strains of *C. parvum*, in both sporozoites and merozoites (Xiao et al., 2025). Approximately 400 genes, 10% of the protein-encoding repertoire, are observed in polycistronic transcripts. The role of polycistronic transcription in gene regulation at either the transcriptional or post-transcriptional level is yet to be explored in *Cryptosporidium* spp. In other eukaryotes, polycistrons have been associated with translational control *via* IRES (Karginov et al., 2017) or leaky ribosomal scanning (Dueñas et al., 2025).

## 6 R-loops

R-loops are DNA:RNA hybrids that can accumulate at specific regions in the genome. They have been associated with gene regulation *via* class switching in immunoglobulins, inhibition of transcription, protection from methylation and open chromatin (Roy et al., 2008; Ginno et al., 2012; Castellano-Pozo et al., 2013; Costantino and Koshland, 2015; D'Souza et al., 2018). R-loops have not yet been experimentally confirmed in *Cryptosporidium* spp., but the abundance of lncRNAs raises the possibility. R-loops have been detected in *P. falciparum* in association with TAREs, reviewed in (Simantov et al., 2022).

## 7 Concluding remarks

The genome sequences for *Cryptosporidium* spp. encode genes for many of the key regulatory proteins and classes of RNAs that have been characterized in other apicomplexans. Yet, given the challenges of working with *Cryptosporidium* spp., the functions of these proteins and RNAs have not been experimentally validated. *Cryptosporidium* spp. also differ from other apicomplexans in that they have retained E2F transcription factors and *C. parvum* has evolved polycistronic transcription. The transcriptional and post-transcriptional regulatory networks that underlie the tight coordination of gene expression in the genus *Cryptosporidium* remain largely unexplored. Recent genetic and technological advancements in *Cryptosporidium* spp. are aiding the community's ability to study gene regulation (Tandel et al., 2023), hopefully moving many of the unknowns into knowns, rapidly. The explosion of new data and insights will facilitate our understanding of gene regulation and biology in *Cryptosporidium* spp. further.

## CRediT authorship contribution statement

**Samantha Gunasekera:** Writing – original draft, Writing – review & editing, Visualization. **Jessica C. Kissinger:** Conceptualization, Writing – original draft, Writing – review & editing.

## Ethical approval

Not applicable.

## Data availability

Not applicable.

## Funding

This work was supported in part by grants from the National Institutes of Health, National Institute of Allergy and Infectious Diseases: R21AI80871 and NIH R01AI148667.

## Declaration of competing interests

The authors declare that they have no known competing financial interests or personal relationships that could have appeared to influence the work reported in this paper.

## References

[bib1] Abrahamsen M.S., Templeton T.J., Enomoto S., Abrahante J.E., Zhu G., Lancto C.A. (2004). Complete genome sequence of the apicomplexan, *Cryptosporidium parvum*. Science.

[bib2] Ammar R., Torti D., Tsui K., Gebbia M., Durbic T., Bader G.D. (2012). Chromatin is an ancient innovation conserved between Archaea and Eukarya. eLife.

[bib3] Attwooll C., Denchi E.L., Helin K. (2004). The E2F family: Specific functions and overlapping interests. EMBO J..

[bib4] Balaji S., Babu M.M., Iyer L.M., Aravind L. (2005). Discovery of the principal specific transcription factors of Apicomplexa and their implication for the evolution of the AP2-integrase DNA binding domains. Nucleic Acids Res..

[bib5] Bankier A.T., Spriggs H.F., Fartmann B., Konfortov B.A., Madera M., Vogel C. (2003). Integrated mapping, chromosomal sequencing and sequence analysis of *Cryptosporidium parvum*. Genome Res..

[bib69] Baptista R.P., Li Y., Sateriale A., Sanders M.J., Brooks K.L., Tracey A., Ansell B.R.E. (2022). Long-read assembly and comparative evidence-based reanalysis of *Cryptosporidium* genome sequences reveal expanded transporter repertoire and duplication of entire chromosome ends including subtelomeric regions. Genome Res..

[bib6] Baptista R.P., Xiao R., Li Y., Glenn T.C., Kissinger J.C. (2025). New T2T assembly of *Cryptosporidium parvum* IOWA II annotated with Legacy-Compatible Gene identifiers. Sci. Data.

[bib7] Bozdech Z., Llinas M., Pulliam B.L., Wong E.D., Jingchun Z., DeRisi J.L. (2003). The transcriptome of the intraerythrocytic developmental cycle of *Plasmodium falciparum*. PLoS Biol..

[bib8] Broadbent K.M., Park D., Wolf A.R., Van Tyne D., Sims J.S., Ribacke U. (2011). A global transcriptional analysis of *Plasmodium falciparum* malaria reveals a novel family of telomere-associated lncRNAs. Genome Biol..

[bib9] Burki F., Roger A.J., Brown M.W., Simpson A.G.B. (2020). The new tree of eukaryotes. Trends Ecol. Evol..

[bib10] Castellano-Pozo M., Santos-Pereira J.M., Rondón A.G., Barroso S., Andújar E., Pérez-Alegre M. (2013). R loops are linked to histone H3 S10 phosphorylation and chromatin condensation. Mol. Cell..

[bib11] Charpentier B., Muller S., Branlant C. (2005). Reconstitution of archaeal H/ACA small ribonucleoprotein complexes active in pseudouridylation. Nucleic Acids Res..

[bib12] Choi S.-W., Keyes M.K., Horrocks P. (2006). LC/ESI-MS demonstrates the absence of 5-methyl-2′-deoxycytosine in *Plasmodium falciparum* genomic DNA. Mol. Biochem. Parasitol..

[bib13] Cohn B., Manque P., Lara A.M., Serrano M., Sheth N., Buck G. (2010). Putative cis-regulatory elements associated with heat shock genes activated during excystation of *Cryptosporidium parvum*. PLoS One.

[bib14] Costantino L., Koshland D. (2015). The Yin and Yang of R-loop biology. Curr. Opin. Cell Biol..

[bib15] D'Souza A.D., Belotserkovskii B.P., Hanawalt P.C. (2018). A novel mode for transcription inhibition mediated by PNA-induced R-loops with a model *in vitro* system. Biochim. Biophys. Acta.

[bib16] De Silva E.K., Gehrke A.R., Olszewski K., León I., Chahal J.S., Bulyk M.L. (2008). Specific DNA-binding by apicomplexan AP2 transcription factors. Proc. Natl. Acad. Sci. USA.

[bib17] Dueñas M.A., Craig R.J., Gallaher S.D., Moseley J.L., Merchant S.S. (2025). Leaky ribosomal scanning enables tunable translation of bicistronic ORFs in green algae. Proc. Natl. Acad. Sci. USA.

[bib18] Eksi S., Morahan B.J., Haile Y., Furuya T., Jiang H., Ali O. (2012). *Plasmodium falciparum* gametocyte development 1 (Pfgdv1) and gametocytogenesis early gene identification and commitment to sexual development. PLoS Pathog..

[bib19] Fazzari M.J., Greally J.M. (2004). Epigenomics: Beyond CpG islands. Nat. Rev. Genet..

[bib20] Fisher O., Siman‐Tov R., Ankri S. (2004). Characterization of cytosine methylated regions and 5‐cytosine DNA methyltransferase (Ehmeth) in the protozoan parasite *Entamoeba histolytica*. Nucleic Acids Res..

[bib21] Ginno P.A., Lott P.L., Christensen H.C., Korf I., Chédin F. (2012). R-loop formation is a distinctive characteristic of unmethylated human CpG island promoters. Mol. Cell..

[bib22] Gissot M., Choi S.-W., Thompson R.F., Greally J.M., Kim K. (2008). *Toxoplasma gondii* and *Cryptosporidium parvum* lack detectable DNA cytosine methylation. Eukaryot. Cell.

[bib23] Grattepanche J.-D., Walker L.M., Ott B.M., Paim Pinto D.L., Delwiche C.F., Lane C.E. (2018). Microbial diversity in the eukaryotic SAR clade: Illuminating the darkness between morphology and molecular data. Bioessays.

[bib24] Hansen J.C. (2002). Conformational dynamics of the chromatin fiber in solution: Determinants, mechanisms, and functions. Annu. Rev. Biophys..

[bib25] Hasan M.M., Mattice E.B., Teixeira J.E., Jumani R.S., Stebbins E.E., Klopfer C.E. (2024). *Cryptosporidium* life cycle small molecule probing implicates translational repression and an Apetala 2 transcription factor in macrogamont differentiation. PLoS Pathog..

[bib26] Iyer L.M., Anantharaman V., Wolf M.Y., Aravind L. (2008). Comparative genomics of transcription factors and chromatin proteins in parasitic protists and other eukaryotes. Int. J. Parasitol..

[bib27] Jing Q., Cao L., Zhang L., Cheng X., Gilbert N., Dai X. (2018). *Plasmodium falciparum* var gene is activated by its antisense long noncoding RNA. Front. Microbiol..

[bib28] Karginov T.A., Pastor D.P.H., Semler B.L., Gomez C.M. (2017). Mammalian polycistronic mRNAs and disease. Trends Genet..

[bib29] Kaur C., Patankar S. (2021). The role of upstream open reading frames in translation regulation in the apicomplexan parasites *Plasmodium falciparum* and *Toxoplasma gondii*. Parasitology.

[bib30] Khorasanizadeh S. (2004). The nucleosome: from genomic organization to genomic regulation. Cell.

[bib31] Li Y., Baptista R.P., Kissinger J.C. (2020). Noncoding RNAs in apicomplexan parasites: An update. Trends Parasitol..

[bib32] Li Y., Baptista R.P., Mei X., Kissinger J.C. (2022). Small and intermediate size structural RNAs in the unicellular parasite *Cryptosporidium parvum* as revealed by sRNA-seq and comparative genomics. Microb. Genom..

[bib33] Li Y., Baptista R.P., Sateriale A., Striepen B., Kissinger J.C. (2021). Analysis of long non-coding RNA in *Cryptosporidium parvum* reveals significant stage-specific antisense transcription. Front. Cell. Infect. Microbiol..

[bib34] Lucky A.B., Wang C., Li X., Chim-Ong A., Adapa S.R., Quinlivan E.P. (2023). Characterization of the dual role of *Plasmodium falciparum* DNA methyltransferase in regulating transcription and translation. Nucleic Acids Res..

[bib35] Matthews J.M., Sunde M. (2002). Zinc fingers - folds for many occasions. IUBMB Life.

[bib36] Mauzy M.J., Enomoto S., Lancto C.A., Abrahamsen M.S., Rutherford M.S. (2012). The *Cryptosporidium parvum* transcriptome during *in vitro* development. PLoS One.

[bib37] Merrick C.J. (2023). Histone lactylation: A new epigenetic axis for host-parasite signalling in malaria?. Trends Parasitol..

[bib38] Ming Z., Gong A.-Y., Wang Y., Zhang X.-T., Li M., Mathy N.W. (2017). Involvement of *Cryptosporidium parvum* Cdg7_FLc_1000 RNA in the attenuation of intestinal epithelial cell migration *via* trans-suppression of host cell SMPD3. J. Infect. Dis..

[bib39] Mitesser V., Simantov K., Dzikowski R. (2024). Time to switch gears: how long noncoding RNAs function as epigenetic regulators in apicomplexan parasites. Curr. Opin. Microbiol..

[bib40] Mullapudi N., Lancto C.A., Abrahamsen M.S., Kissinger J.C. (2007). Identification of putative cis-regulatory elements in *Cryptosporidium parvum* by *de novo* pattern finding. BMC Genom..

[bib41] Nakamoto M.A., Lovejoy A.F., Cygan A.M., Boothroyd J.C. (2017). mRNA pseudouridylation affects RNA metabolism in the parasite *Toxoplasma gondii*. RNA.

[bib42] Oberstaller J., Joseph S.J., Kissinger J.C. (2013). Genome-wide upstream motif analysis of *Cryptosporidium parvum* genes clustered by expression profile. BMC Genom..

[bib43] Oberstaller J., Pumpalova Y., Schieler A., Llinás M., Kissinger J.C. (2014). The *Cryptosporidium parvum* ApiAP2 gene family: Insights into the evolution of apicomplexan AP2 regulatory systems. Nucleic Acids Res..

[bib44] Possemiers H., Vandermosten L., Van den Steen P.E. (2021). Etiology of lactic acidosis in malaria. PLoS Pathog..

[bib45] Prouse M.B., Campbell M.M. (2012). The interaction between MYB proteins and their target DNA binding sites. Biochim. Biophys. Acta.

[bib46] Radke J.R., Behnke M.S., Mackey A.J., Radke J.B., Roos D.S., White M.W. (2005). The transcriptome of *Toxoplasma gondii*. BMC Biol..

[bib47] Richmond T.J., Davey C.A. (2003). The structure of DNA in the nucleosome core. Nature.

[bib48] Rider S.D., Zhu G. (2009). An apicomplexan ankyrin-repeat histone deacetylase with relatives in photosynthetic eukaryotes. Int. J. Parasitol..

[bib49] Roy D., Yu K., Lieber M.R. (2008). Mechanism of R-loop formation at immunoglobulin class switch sequences. Mol. Cell Biol..

[bib50] Sautel C.F., Cannella D., Bastien O., Kieffer S., Aldebert D., Garin J. (2007). SET8-mediated methylations of histone H4 lysine 20 mark silent heterochromatic domains in apicomplexan genomes. Mol. Cell Biol..

[bib51] Sawant M., Benamrouz-Vanneste S., Meloni D., Gantois N., Even G., Guyot K. (2022). Putative SET-domain methyltransferases in *Cryptosporidium parvum* and histone methylation during infection. Virulence.

[bib52] Schwarz D., Lourido S. (2023). The multifaceted roles of Myb domain-containing proteins in apicomplexan parasites. Curr. Opin. Microbiol..

[bib53] Siegel T.N., Hon C.-C., Zhang Q., Lopez-Rubio J.-J., Scheidig-Benatar C., Martins R.M. (2014). Strand-specific RNA-Seq reveals widespread and developmentally regulated transcription of natural antisense transcripts in *Plasmodium falciparum*. BMC Genom..

[bib54] Simantov K., Goyal M., Dzikowski R. (2022). Emerging biology of noncoding RNAs in malaria parasites. PLoS Pathog..

[bib55] Sterner D.E., Berger S.L. (2000). Acetylation of histones and transcription-related factors. Microbiol. Mol. Biol. Rev..

[bib56] Strahl B.D., Allis C.D. (2000). The language of covalent histone modifications. Nature.

[bib57] Tandel J., English E.D., Sateriale A., Gullicksrud J.A., Beiting D.P., Sullivan M.C. (2019). Life cycle progression and sexual development of the apicomplexan parasite *Cryptosporidium parvum*. Nat. Microbiol..

[bib58] Tandel J., Walzer K.A., Byerly J.H., Pinkston B., Beiting D.P., Striepen B. (2023). Genetic ablation of a female-specific Apetala 2 transcription factor blocks oocyst shedding in *Cryptosporidium parvum*. mBio.

[bib59] Templeton T.J., Iyer L.M., Anantharaman V., Enomoto S., Abrahante J.E., Subramanian G.M. (2004). Comparative analysis of Apicomplexa and genomic diversity in eukaryotes. Genome Res..

[bib60] Waller R.F., Carruthers V.B. (2024). Adaptations and metabolic evolution of myzozoan protists across diverse lifestyles and environments. Microbiol. Mol. Biol. Rev..

[bib61] Walzer K.A., Tandel J., Byerly J.H., Daniels A.M., Gullicksrud J.A., Whelan E.C. (2024). Transcriptional control of the *Cryptosporidium* life cycle. Nature.

[bib62] Wang Y., Gong A.-Y., Ma S., Chen X., Li Y., Su C.-J. (2016). Delivery of parasite RNA transcripts into infected epithelial cells during *Cryptosporidium* infection and its potential impact on host gene transcription. J. Infect. Dis..

[bib63] Wang Y., Gong A.-Y., Ma S., Chen X., Strauss-Soukup J.K., Chen X.-M. (2017). Delivery of parasite Cdg7_Flc_0990 RNA transcript into intestinal epithelial cells during infection suppresses host cell gene transcription through epigenetic mechanisms. Cell. Microbiol..

[bib64] Wei H., Jiang S., Chen L., He C., Wu S., Peng H. (2017). Characterization of cytosine methylation and the DNA methyltransferases of *Toxoplasma gondii*. Int. J. Biol. Sci..

[bib65] Xiao R., Baptista R.P., Agyabeng-Dadzie F., Li Y., Dong Y., Schmitz R.J. (2025). Deciphering transcription in *Cryptosporidium parvum*: Polycistronic gene expression and chromatin accessibility. bioRxiv.

[bib66] Yasukawa H., Yagita K. (2010). Silent information regulator 2 proteins encoded by *Cryptosporidium* parasites. Parasitol. Res..

[bib67] Zhang D., Tang Z., Huang H., Zhou G., Cui C., Weng Y. (2019). Metabolic regulation of gene expression by histone lactylation. Nature.

[bib68] Zhang H., Guo F., Zhu G. (2015). *Cryptosporidium* lactate dehydrogenase is associated with the parasitophorous vacuole membrane and is a potential target for developing therapeutics. PLoS Pathog..

